# Obtaining interactions among science, technology, and research policy for developing an innovation strategy: A case study of supercapacitors

**DOI:** 10.1016/j.heliyon.2022.e10721

**Published:** 2022-09-21

**Authors:** Chika Ishii, Kimitaka Asatani, Ichiro Sakata

**Affiliations:** a Cisco Systems G.K., Tokyo, Japan; b Graduate School of Engineering, The University of Tokyo, Tokyo, Japan

**Keywords:** Bibliometrics, Technology maturity, Innovation strategy, Network science

## Abstract

Comprehensive observations of science, technology, and research policy transactions are important for developing an innovation strategy. We propose a new method that combines the academic landscape and matrix analysis to understand the relationships among activities of three aspects of the technological landscape: science, technology, and research policy. First, we divided academic research into 28 knowledge domains by clustering a citation network of scientific papers. Next, we developed a new matrix classifying them into three groups: “mature technology,” “intermediate technology,” and “emerging technology.” The results showed that research domains in “emerging technology” showed a high rate of patent increase, indicating that they were commercializing rapidly. Finally, we identified the group that each country focused on, and this result reflected the countries' research policies. China and Singapore showed high rates, whereas Japan, France, and Germany had low values. This result reflects countries’ research policies and implies that specialty research areas differed by country. As above, our research result implies that academia, industry, and government have paid attention to knowledge domains in “emerging technology” and these are important for creating innovation. A supercapacitor, also known as an electric double layer capacitor or ultracapacitor, was selected as an example in our method. This research could help academic researchers, industrial companies, and policymakers in developing innovation strategies.

## 1 Introduction

In recent years, science and technology have been developing at a remarkable pace, and the number of research papers has continued to increase exponentially [1]. In 2016, approximately 2.2 million papers, an enormous number, were published in one year [2]. However, the rapid increase in the number of papers has caused issues such as the obscuring of the overall picture of the scientific field. The rapid increase in scientific papers has made understanding the entire picture of the scientific field difficult even for experts [3].

To deal with this issue, we use the approach of conducting a structural overview of knowledge using the visualization of vast volumes of academic papers and bibliographic information [4, 5, 6]. This visualization is used to understand the current situation and predict the knowledge domains that will grow in the future [7]. For researchers with limited research resources, it is important to predict knowledge areas that will grow in the future as it enables them to select research topics with a high potential of leading to innovation creation.

Previous methods consider that knowledge domains with a low level of maturity have room for innovation creation [8, 9]. Although there are various metrics to assess the maturity of knowledge domains, in most cases, it has been evaluated using one variable, such as average publication year and the rate of growth in publication [10, 11, 12, 13]. However, there may be some knowledge domains that have appeared recently and have already published an enormous number of papers, given that the number of publications has been increasing dramatically.

The visualization of academic articles is also used for developing an innovation strategy. As the triple helix of academia-industry-government relations is important for creating innovation, observing the interactions among these actors is necessary [14]. Earlier studies have reported methods for observing science, technology, and research policy as follows:

A landscape of science can be obtained by visualizing a high number of publications, for instance, a global map of science, science overlay maps, VOS mapping, and science maps [15, 16, 17, 18, 19]. In these methods, publications are classified into knowledge domains with similar subjects by citation network clustering, which assists in grasping the entire picture of the research area. This is based on the theory that citing and cited papers have similarities or at least related research subjects [20]. The academic landscape, a landscape of science, provides us with a more detailed micro-view of knowledge domain visualization than prior research because it analyzes datasets related to a specific research area [21, 22, 23].

A landscape of technology can be realized by comparing the structure of the citation networks of patents with that of scientific publications. Scientific papers typically report the results of scientific research, while patents describe the invention of a new product, process, or machine and play an important role in commercializing them. Papers and patents interact with each other, as 80% of cited publications link forward to future patents, and 61% of patents link backward to earlier publications [24]. Various uses of linkages between academic articles and patents have been reported such as the detection of the technology opportunity, understanding university-industry-government interaction, assessing innovation, and making high-quality roadmaps [25, 26, 27, 28, 29]. As academic papers and patents have the abovementioned interactions, knowledge domains that exist in academic research but not in industry can be regarded as opportunities for industrial commercialization [30, 31]. This theory has been used to discover potential opportunities for industrial commercialization.

An outcome of research policy can be obtained through an international comparison of research tendencies [19, 32]. The superior or inferior research domains of each country were extracted by analyzing the publications' characteristics. In the case of the science map, technology areas were split along two axes: continuity and involvement with other research areas. Countries’ research minds were compared using a ratio of areas. The research areas were classified into four groups: “continent type,” “island type,” “peninsula type,” and “small island type.” Research areas that had continuity with the previous map were categorized as “continent type,” wherein research areas were strongly related to others, and “island type,” which included research areas having more fragile relationships with others. Research areas that had no continuity with the past map were classified as “peninsula type,” which filled the peripheral positions of “continent type” and “small island type,” which included research areas that had weaker relationships with others. One of the results revealed that because Japan accounts for a small share of “small-island type,” Japanese researchers should focus on unique research.

Observing the relationships among key actors–science, technology, and research policy–is necessary for considering an innovation strategy. However, in most cases, earlier bibliometric analyses for developing an innovation strategy have only reported views on university-industry collaboration [33, 34, 35, 36, 37]. Additionally, previous bibliometric studies on the triple helix have focused on the strength of industry-academia-government relationships [38, 39]. Given that specific technical areas were not discussed in these studies, finding important knowledge domains for developing an innovation strategy is difficult. The main purpose of this study is to propose a new method to find important knowledge domains for developing an innovation strategy and analyze the relationships among three actors–science, technology, and research policy. In this study, we developed a new matrix to evaluate the maturity of knowledge domains with two metrics because using one variable may not be able to accurately assess the maturity of knowledge domains. Our method identifies developing knowledge domains of scientific research, and the origin of innovation, and estimates its impact on industry and government to analyze the interaction among academia, industry, and government.

## 2 Method

### 2.1 The outline of our method

The analysis process consisted of three steps. First, we created academic landscapes and grasped the knowledge domains that exist in scientific research [21, 22, 23]. Next, we categorized them into three groups based on their maturity, namely, “mature technology,” “intermediate technology,” and “emerging technology,” using the matrix. Finally, we calculated the share of each technology area by country to compare international research tendencies.

### 2.2 Identifying knowledge domains

We conducted the following citation network analysis to identify the knowledge domains.

First, we collected academic papers on the target research area. We obtained academic articles and citation data from the Science Citation Index and the Social Science Citation Index collected by the Institute for Science Information. We used Web of Science (WoS), an online subscription-based scientific citation indexing service maintained by Clarivate Analytics, to access these databases. This is because we needed to extract scientific papers published over a wide range of years, and WoS has a wider range of journal publication years than other databases.

Second, the citation network was converted to a non-weighted non-directed network, as shown in Figure 1. There are three types of citation networks: direct citation, co-citation, and bibliographic coupling. Direct citation is defined as the edge between citing and cited publications. Co-citation illustrates the link between two papers cited by the same document [40]. Bibliographic coupling describes the edge between two publications citing the same paper [41]. We adopted a direct citation network because it was shown to be the best suited to identify emerging clusters [42, 43, 44].

**Figure 1:**
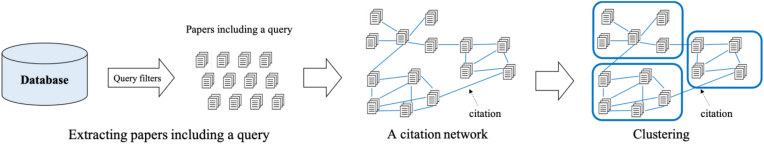
Methods for extracting knowledge domains.

Third, the network was divided into clusters using the topological clustering method to maximize modularity (Q) invented by Newman [45, 46]. A high value of Q means an appropriate division of the network, in other words, the remaining dense edges within clusters and the elimination of the sparse links between clusters. This theory was based on the following motivation. The few edges linked between communities can be regarded as forming “bottlenecks” between them, and traffic that flows through the network will have to move along at least one of these bottleneck edges if it wants to pass from one community to another. Thus, if we examine a model of traffic on the network and search for links with the highest traffic, we should be able to detect the edges between the communities. Removing these should divide the network into reasonable communities. Q is the fraction of the links that fall within the given groups minus the anticipated fraction if links were offered randomly. Starting with a state in which each node is a single member of one of the clusters, we repeatedly connect clusters together in pairs, picking the join-up at each step that results in the greatest increase (or smallest decrease) in Q. Making cuts through a topology at different degrees divides the network into larger or smaller number of clusters and we can find the best cut by searching for the maximal value of Q. According to previous studies, this method is reasonable for extracting knowledge domains [47]. In this study, we clustered a citation network three times to obtain sufficiently small clusters to accurately understand the knowledge domains. Q is calculated as following:

$$Q=\;\overset{M}{\underset{s=1}{\sum }}\left[\frac{l_{s}}{l}-{\left(\frac{d_{s}}{2l}\right)}^{2}\right]\;$$

*M*: number of clusters, *s*: the given cluster, *l*: the total number of edges in the network, *l*_*s*_: the links between two nodes within cluster s, *d*_*s*_: the total number of internal links of cluster.

Finally, we labeled the major clusters to understand the content contained in them. Given that describing words appear more often in the knowledge domain than in others, we named each cluster based on keywords with a high term frequency-inverse document frequency (TF-IDF) value [48]. TF-IDF is the product of TF and IDF, where TF is the term frequency and IDF is the inverse document frequency. TF, IDF, and TF-IDF can be calculated using the following equations:

$$TF\left(t,\;d\right)=\;log\;\left(1\;+\;f_{t,\;d}\right)$$

$$IDF\left(t,\;D\right)=\;log\;\frac{\left|D\right|}{\left|d\;\in \;D\;:\;t\;\in \;d\right|}$$

TF−IDF(t,d,D)=TF(t,d)×IDF(t,D)

*t*: term, *d*: document, *D*: total number of documents.

### 2.3 Evaluating the maturity of knowledge domains

As we mentioned, there are various metrics to assess the maturity of research areas, and we chose the average publication year and the number of nodes from them. According to the maturity assessment framework offered by Keathley-Herring, the average publication year and the number of nodes are defined as the publication quantity [10]. We regarded them as an appropriate metric in this study because they can quantify the characteristics of a group including a large number of publications. Other metrics such as year of first publication and some unique outlets focus on a part of publications, while others like the strength of evidence for reliability/validity and several disciplines represented in dedicated journals are difficult to quantify in the case of many papers. Previous studies have evaluated the maturity of knowledge domains including many scientific articles with average publication year and the number of nodes [49, 50, 51].

**Figure 2:**
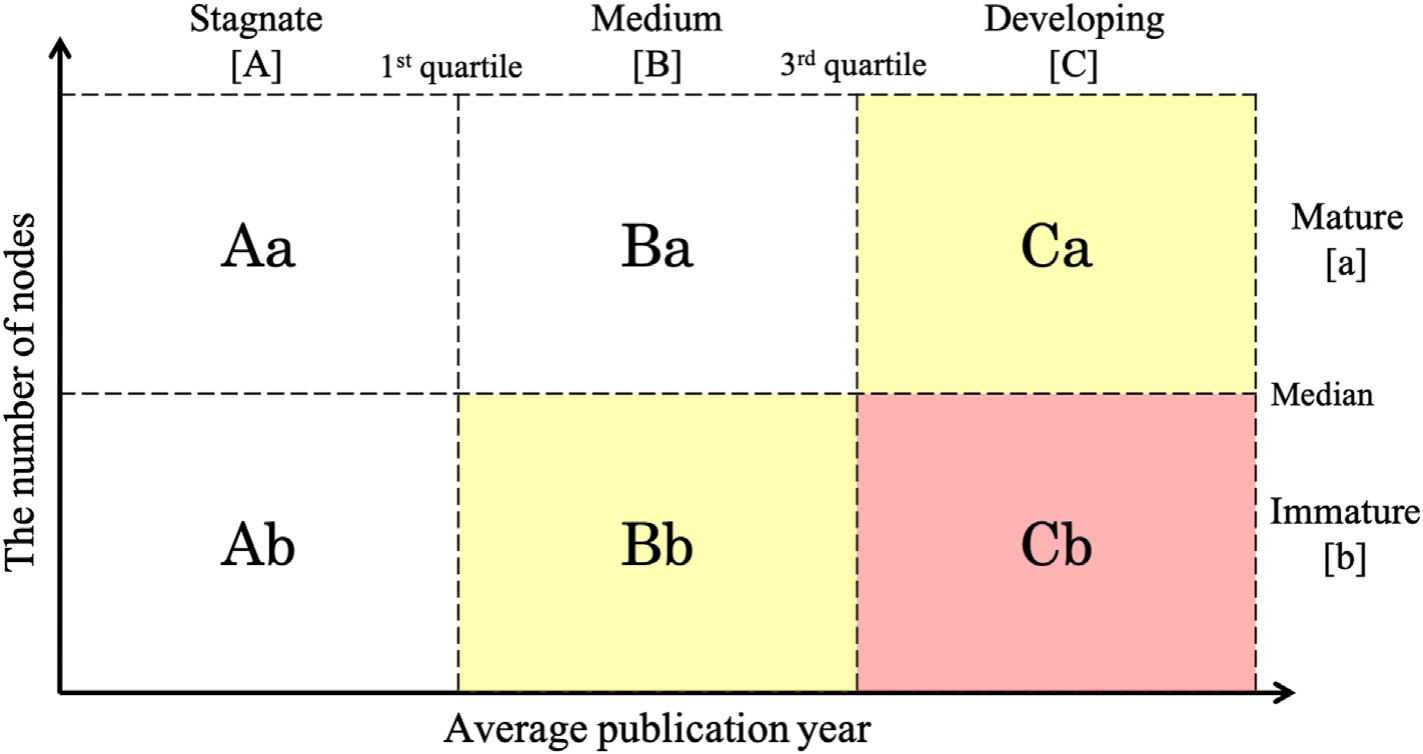
The new matrix for evaluating the maturity of knowledge domains (white, yellow, and red area represent “mature technology,” “intermediate technology,” and “emerging technology,” respectively).

The matrix was developed to evaluate the maturity of the knowledge domains, as follows (Figure 2). The X- and Y-axis denote the average publication year of the cluster and the number of nodes, respectively. The X-axis is separated into three parts: stagnate [A] (the lowest 25% of data), medium [B] (between 25% and 75%), and developing [C] (the highest 25% of data). The Y-axis is split into two parts: mature [a] (the upper half) and immature [b] (the bottom half). Thus, we divided the graph into six areas: Aa, Ab, Ba, Bb, Ca, and Cb. We classified knowledge domains into three technology maturity classes as follows: those in Aa, Ab, Ba into “mature technology,” those in Bb, Ca into “intermediate technology,” and those in Cb into “emerging technology.”

To understand the speed of commercialization of knowledge domains, we counted the number of patents up to 2018/11/28 and 2021/11/28 and calculated the percentage increase between three years in each knowledge domain. We searched for patents in the World Intellectual Property Organization (WIPO) IP Portal, which provides access to international and national patents [52]. The WIPO IP Portal has two search modes: “simple search,” and “advanced search.” The following letter and options were used: "field” (in which patent includes queries) is front page, “offices” (patent issuing country or organization) is all, “language” (in which patent is written) is English, “stemming” is true, “single family member” (displaying documents belonging to the same patent family together) is false, “include NPL (non-patent literature)” is false.

There are three different types of patents, namely, utility patents, design patents, and plant patents [53], that may be granted to whoever invents a new matter or article of manufacture, design of an article of manufacture, and a variety of a plant, respectively. The most common patent applications documented at United States Patent and Trademark Office (USPTO) are for utility patents. It has been reported that 47% of patents were granted, and 61% of the granted patents were commercialized, based on which we note that commercializing patents is difficult [54].

### 2.4 International comparison of research tendency

We counted the number of publications and patents of each technology area: “mature technology,” “intermediate technology,” and “emerging technology,” by county and calculated the share of each technology area. We examined the top ten countries with the most number of publications. To understand the research trends of countries, we compared countries' values and assessed each country's technology area focus.

### 2.5 Supercapacitors

**Figure 3:**
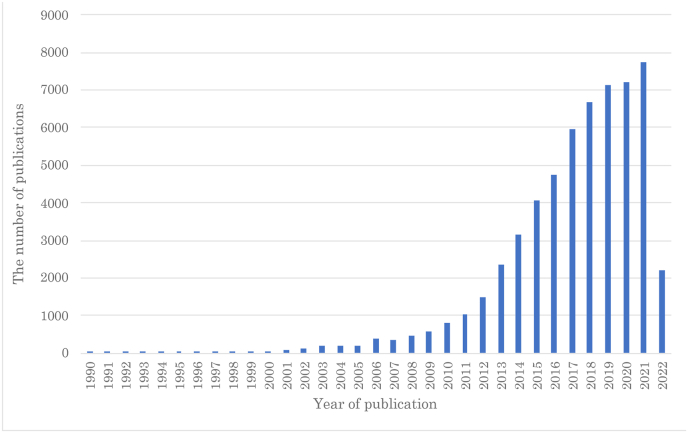
The number of publications including the query on WoS.

A supercapacitor (SC) is a capacitor that uses an electric double layer to charge/discharge with a capacitance value much higher than that of other capacitors. It can accept and deliver charge much faster than other batteries owing to its high capacity and ability to endure many more charge-discharge cycles than other rechargeable batteries [55]. In this study, we selected SC as a research target because it is popular in academic research, industrial businesses, and technology policies. From the academic perspective, the number of publications including one of the following keywords—“supercapacit∗,” “electrical double layer capacito∗,” “edlc,” or “ultracapcit∗” (the asterisk∗ represents a wildcard that can help find the appropriate results)—on WoS has been rapidly increasing with 7,732 such papers published in 2021 (Figure 3). From the industrial perspective, the global supercapacitor market has been growing continuously and reached 16 billion US dollars in 2015 [56]. Finally, from a policymaker perspective, countries across the world are investing in SC development. For example, the National Natural Science Foundation of China launched 338 national projects related to graphene-based SC [57].

## 3 Results

### 3.1 Collecting academic papers on SC

Highly cited academic articles on SC state that the electric double layer capacitor is also known as EDLC, supercapacitor, or ultracapacitor [58, 59, 60, 61]. Therefore, we set the following queries for covering the research field of SC: “supercapacit∗,” “electrical double layer capacito∗,” “edlc,” and “ultracapcit∗“, and select “all fields” as research fields that enable search papers including selected keywords in title, abstract, or keywords. A total of 35,068 papers were collected (up to 2018/11/28). Publications not linked to any others were eliminated in this step because we considered papers having no citations with any others to be irrelevant to the main topic. We analyzed the largest connected component, which accounted for approximately 96% of the collected papers (33,750 out of 35,068 papers).

The number of the extracted papers per publication year has been increasing continuously as shown in Figure S1. The average number of authors per publication is 5.37. The average and the median number of citations by extracted papers per article is 37.5 and 13, respectively. The most cited paper is written by Simon, which has been cited 8,577 times [62]. Electrochimica Acta, which specializes in electrochemicals, is the journal with the most number of publications (2,720 papers). The top country is China, which published 15,022 papers. The top institution is the Chinese Academy of Science, which issued 1,956 articles. The top ten journals, countries, and research institutes with the largest number of publications are shown in Table 1. China and the Chinese Academy of Science have the highest number of publications among countries and institutions, respectively.

**Table 1:** The number of publications of the top 10 journals/countries/institutes.

Ranking	Top 10 (The number of publications)		
	Journal	Country	Institute
1	Electrochimica Acta (2,720)	China (15,022)	Chinese Academy of Sciences (1,956)
2	Journal of Power Sources (1,758)	USA (3,558)	Nanyang Technological University (650)
3	Journal of Materials Chemistry A (1,757)	South Korea (2,671)	Tsinghua University (631)
4	RSC Advances (1,623)	India (2,216)	University of Chinese Academy of Sciences (489)
5	ACS Applied Materials & Interfaces (972)	Japan (1,293)	Fudan University (456)
6	Carbon (793)	Australia (970)	Huazhong University of Science and Technology (401)
7	Journal of The Electrochemical Society (748)	Singapore (886)	Chongqing University (389)
8	Journal of Alloys and Compounds (711)	Germany (849)	Jilin University (383)
9	Applied Surface Science (542)	France (778)	Zhejiang University (382)
10	Materials Letters (533)	Taiwan (774)	Harbin Institute of Technology (368)

### 3.2 Identifying knowledge domains

The citation network of the collected papers was split into 76 clusters by clustering. The number of nodes in each cluster was 12,652, 12,161, 7,683, 670…2, in descending order. We considered the 1^st^, 2^nd^, and 3^rd^ largest clusters (each cluster was named #1, #2, and #3) because the number of nodes in each cluster became insignificant after the 4^th^ cluster.

We conducted an in-depth clustering of the academic journals into clusters #1, #2, and #3 to more deeply understand the clusters. We defined the b^th^ largest sub-cluster out of sub-clusters derived from #a as #a-b. We considered #1-1, #1-2, #1-3, #2-1, #2-2, #2-3, #3-1, #3-2, and #3-3, because the number of nodes in each cluster became insignificant after the 4^th^ sub-cluster.

We conducted an in-depth clustering of the academic papers into sub-clusters. Subsequently, we acquired 28 sub-sub-clusters that were big enough to be analyzed. We defined the c^th^ largest sub-sub-cluster out of the sub-sub-clusters derived from #a-b as #a-b-c. We labelled the sub-sub-clusters based on their descriptions (Table 2). The sub-sub-clusters were named as follows: #1-1-1 activated carbon, #1-1-2 MnO_2_, #1-1-3 polymer electrolyte, #1-2-1 nitrogen doped activated carbon, #1-2-2 biomass carbon, #1-2-3 electrospun carbon nanofiber (ECNF), #1-3-1 application development, #1-3-2 pore size in mesoporous carbon, #1-3-3 ionic liquid, #2-1-1 nitrogen doped graphene, #2-1-2 poly-aniline/graphene (PANi/G), #2-1-3 carbon nanotube (CNT), #2-2-1 flexible SC, #2-2-2 micro SC, #2-2-3 wearable SC, #2-2-4 asymmetric SC, #2-3-1 conducting polymer/CNT, #2-3-2 PANi/CNT, #2-3-3 MoS_2,_ #3-1-1 MnO_2_, #3-1-2 NiCo_2_O_4_, #3-1-3 MCo_2_O_4_ (M = Zn, Mn, or Cu), #3-2-1 NiO, #3-2-2 V_2_O_5_, #3-2-3 layered double hydroxides (LDH), #3-3-1 metal organic framework (MOF), #3-3-2 Ni_3_N_2_, and #3-3-3 NiCo_2_S_4_. In this study, we considered these sub-sub-clusters for knowledge domains.

**Table 2:** Keyword of each sub-sub-cluster.

Sub-sub-cluster	Keyword	TF-IDF	Sub-sub-cluster	Keyword	TF-IDF	Sub-sub-cluster	Keyword	TF-IDF
#1-1-1	carbon	0.00266	#1-3-3	redox	0.00313	#2-3-1	carbon nanotube	0.00164
	activated	0.00232		ionic liquid	0.00297	#2-3-2	PANi	0.00630
#1-1-2	MnO_2_	0.00654	#2-1-1	nitrogen	0.00231		carbon nanotube	0.00132
#1-1-3	polymer electrolyte	0.00714		doped graphene	0.00156	#2-3-3	MoS_2_	0.01304
#1-2-1	nitrogen doped	0.00192	#2-1-2	PANi	0.00397	#3-1-1	MnO_2_	0.00534
	mesoporous carbon	0.00134		composite	0.00245	#3-1-2	NiCo_2_O_4_	0.00644
#1-2-2	activated carbon	0.00169	#2-1-3	carbon nanotube	0.00212	#3-1-3	ZnCo_2_O_4_	0.00411
	derived	0.00160	#2-2-1	flexible	0.00200		MnCo_2_O_4_	0.00347
	lignin	0.00154		stretchable	0.00178		CuCo_2_O_4_	0.00273
#1-2-3	carbon nanofibers	0.00176	#2-2-2	micro supercapacitors	0.00233	#3-2-1	NiO	0.00607
	electrospinning	0.00149	#2-2-3	fiber	0.00683	#3-2-2	V_2_O_5_	0.00419
#1-3-1	power	0.00463		yarn	0.00360	#3-2-3	LDH	0.00534
	system	0.00439		wearable	0.00243	#3-3-1	metal organic framework	0.00224
	control	0.00422		textile	0.00210		MOFs	0.00167
#1-3-2	carbon	0.00393	#2-2-4	asymmetric	0.00226	#3-3-2	Ni_3_S_2_	0.00301
	pore	0.00376	#2-3-1	PEDOT	0.00358		sulfide	0.00232
	size	0.00248		polypyrrole	0.00243	#3-3-3	NiCo_2_S_4_	0.00730

We classified the knowledge domains into three categories according to their keywords: “electrode materials,” “application,” and “others.” The number of knowledge domains categorized as “electrode materials,” “application,” and “others,” were 22, 4, and 2, respectively. The “electrode materials” category included #1-1-1 activated carbon, #1-1-2 MnO_2_, #1-1-3 polymer electrolyte, #1-2-1 nitrogen doped activated carbon, #1-2-2 biomass carbon, #1-2-3 ECNF, #2-1-1 nitrogen doped graphene, #2-1-2 PANi/G, #2-1-3 CNT, #2-3-1 conducting polymer/CNT, #2-3-2 PANi/CNT, #2-3-3 MoS_2_, #3-1-1 MnO_2_, #3-1-2 NiCo_2_O_4_, #3-1-3 MCo_2_O_4_, #3-2-1 NiO, #3-2-2 V_2_O_5_, #3-2-3 LDH, #3-3-1 MOF, #3-3-2 Ni_3_N_2_, and #3-3-3 NiCo_2_S_4_. The “application” category included #1-3-1 application development, #2-2-1 flexible SC, #2-2-2 micro SC, #2-2-3 wearable SC, and #2-2-4 asymmetric SC. The “others” category included #1-3-2 pore size in mesoporous carbon, and #1-3-3 ionic liquid.

### 3.3 Evaluating maturity of knowledge domains

The classification results of the sub-sub-clusters are shown in Table 3. Six knowledge domains, #2-2-3 wearable SC, #2-2-4 asymmetric SC, #2-3-3 MoS_2_, #3-1-3 MCo_2_O_4_, #3-3-1 Ni_3_S_2_, and #3-3-3 NiCo_2_S_4_, were classified as “emerging technology.” The following ones were defined as “intermediate technology”: #1-2-2 biomass carbon, #3-1-2 NiCo_2_O_4_, #3-2-1 NiO, #3-2-2 V_2_O_5_, #3-2-3 LDH, #3-3-2 Ni_3_N_2_. The other 16 knowledge domains were categorized as “mature technology.” Most of the knowledge domains in “emerging technology” are related to research on metals like oxides/sulfides and MOF. In contrast, many knowledge domains in “mature technology” have focused on carbon materials such as activated carbon and graphene.

**Table 3:** Classification of the knowledge domains by classes.

Maturity class	Area	Knowledge domain
Mature technology	Aa	#1-1-1 activated carbon, #1-1-2 MnO_2_, #1-3-1 application development, #2-3-1 conducting polymer/CNT
	Ab	#1-1-3 polymer electrolyte, #1-3-3 ionic liquid, #2-3-2 PANi/CNT
	Ba	#1-2-1 nitrogen doped activated carbon, #1-2-3 ECNF, #1-3-2 pore size in mesoporous carbon, #2-1-1 nitrogen doped graphene, #2-1-2 PANi/G, #2-1-3 CNT, #2-2-1 flexible SC, #2-2-2 micro SC, #3-1-1 MnO_2_
Intermediate technology	Bb	#3-1-2 NiCo_2_O_4_, #3-2-1 NiO, #3-2-2 V_2_O_5_, #3-2-3 LDH, #3-3-2 Ni_3_S_2_
	Ca	#1-2-2 biomass carbon
Emerging technology	Cb	#2-2-3 wearable SC, #2-2-4 asymmetric SC, #2-3-3 MoS_2_, #3-1-3 MCo_2_O_4_, #3-3-1 MOF, #3-3-3 NiCo_2_S_4_

The number of patents up to 2018/11/28 and 2021/11/28 (hereinafter referred to as “A” and “B,” respectively) and the percentage increase between three years ($\frac{B-A}{A}$) in each knowledge domain are shown in Table 4. One of the knowledge domains in “emerging technology,” #3-3-1 MOF, showed the largest increase rate of patents (478.9%). Others, #2-2-3 wearable SC, and #3-3-3 NiCo_2_S_4_, also showed higher values (178.6%, 293.3%). In contrast, knowledge domains in “mature technology” tended to have lower rates. For example, the increase rate of patents of #1-1-1 activated carbon, #1-1-3 polymer electrolyte, and #1-2-3 ECNF were 20.8%, 16.0%, and 0.0%, respectively. Domains in “intermediate technology” tended to show values higher than “mature technology” and lower than “emerging technology,” and, notably, #3-3-2 Ni_3_N_2_ showed a high value (263.6%).

**Table 4:** The number and percentage increase of patents in each class.

Maturity class	Knowledge domain	Query ※Patents were searched with queries below adding “AND (supercapacit∗ OR (double layer capacit∗))”	A	B	$\frac{B-A}{A}$ [%]
Mature technology	#1-1-1	activated carbon	1,262	1,525	20.8%
	#1-1-2	“manganese oxide” OR MnO_2_	122	225	84.4%
	#1-1-3	“polymer electrolyte”	156	181	16.0%
	#1-2-1	nitrogen AND doped AND mesoporous AND carbon	9	23	155.6%
	#1-2-3	“carbon nanofiber∗” AND electrospinning	4	4	0.0%
	#1-3-1	power AND control AND system	395	610	54.4%
	#1-3-2	size AND pore AND carbon	139	195	40.3%
	#1-3-3	“ionic liquid” AND (redox OR based)	34	57	67.6%
	#2-1-1	nitrogen AND doped AND graphene	60	117	95.0%
	#2-1-2	(polyaniline OR PANi) AND graphene	56	96	71.4%
	#2-1-3	“carbon nanotub∗” OR NT	406	635	56.4%
	#2-2-1	flexible AND carbon	148	304	105.4%
	#2-2-2	“micro supercapacit∗” OR MSC	58	97	67.2%
	#2-3-1	(PEDOT OR (polypyrrole OR PPy)) AND (“carbon nanotub∗” OR CNT)	12	19	58.3%
	#2-3-2	(polyaniline OR PANi) AND (“carbon nanotub∗” OR CNT)	19	41	115.8%
	#3-1-1	(manganese AND (dioxide OR oxide)) OR MnO_2_	250	419	67.6%
Intermediate technology	#1-2-2	(lignin OR derived) AND activated AND carbon	22	54	145.5%
	#3-1-2	“nickel cobaltite” OR NiCo_2_O_4_	31	55	77.4%
	#3-2-1	“nickel oxide” OR NiO	63	109	73.0%
	#3-2-2	“vanadium oxide” OR V_2_O_5_	12	19	58.3%
	#3-2-3	layered AND double AND hydroxid∗	20	52	160.0%
	#3-3-2	“nickel sulfide” OR Ni_3_S_2_	11	40	263.6%
Emerging technology	#2-2-3	(fiber OR yarn OR textile) AND wearable	14	39	178.6%
	#2-2-4	asymmetri∗	189	296	56.6%
	#2-3-3	“molybdenum disulfide” OR MoS_2_	36	77	113.9%
	#3-1-3	MnCo_2_O_4_ OR ZnCo_2_O_4_ OR CuCo_2_O_4_	6	13	116.7%
	#3-3-1	“metal organic framework” OR MOF	19	110	478.9%
	#3-3-3	(nickel AND cobalt AND sulfid∗) OR NiCo_2_S4	15	59	293.3%

### 3.4 International comparison of research tendency

To understand the research trends of countries, we calculated and compared the following two values of the ten countries.

First, we counted the number of extracted academic articles published by 2018/11/28 in each technology class and calculated the ratio of classes by country (Table 5). The number of publications in the top five countries is as follows: China (28,232), the USA (6,444), South Korea (5,329), India (3,863), and Japan (2,158). Other countries include France, Taiwan, Germany, Australia, and Singapore, which published approximately 1500 scientific papers. As China accounts for 80.5% of extracted papers (28,232 out of 35,068), the number of publications is concentrated in China. Comparing the percentage of “emerging technology,” China and Singapore have a higher ratio of “emerging technology,” 18.3%, and 22.1%, respectively. Japan, France, and Germany have a lower ratio: 4.5%, 1.1%, and 4.4%, respectively. The others including the USA, South Korea, India, and Australia have intermediate values: 9.6%, 12.6%, 11.9%, and 11.0%, respectively.

**Table 5:** The number of publications in each class by countries.

Country	The number of publications (The percentage of publications in each technology by country)			
	Mature technology	Intermediate technology	Emerging technology	Total
China	14,124 (50.0%)	8,942 (31.7%)	5,166 (18.3%)	28,232 (100.0%)
USA	3,436 (53.3%)	2,388 (37.1%)	620 (9.6%)	6,444 (100.0%)
South Korea	3,050 (57.2%)	1,610 (30.2%)	669 (12.6%)	5,329 (100.0%)
India	2,151 (55.7%)	1,254 (32.5%)	458 (11.9%)	3,863 (100.0%)
Japan	1,371 (63.5%)	690 (32.0%)	97 (4.5%)	2,158 (100.0%)
France	965 (62.0%)	574 (36.9%)	17 (1.1%)	1,556 (100.0%)
Taiwan	940 (61.1%)	478 (31.1%)	121 (7.9%)	1,539 (100.0%)
Germany	822 (54.0%)	639 (42.0%)	61 (4.0%)	1,522 (100.0%)
Australia	785 (56.0%)	463 (33.0%)	154 (11.0%)	1,402 (100.0%)
Singapore	658 (46.9%)	434 (31.0%)	310 (22.1%)	1,402 (100.0%)

Second, we counted the number of patents published from 2018/11/29 to 2021/11/28 and calculated the ratio of classes by country (except for Taiwan due to data unavailability), and the result is shown in Table 6. Focusing on the number of patents of each country, the number of patents is more biased than academic publications. China accounts for the 89.0% of all patents (1084 out of 1,233 patents). The USA, South Korea, and India account for 60, 44, and 37 patents, respectively, while Australia has 6 patents. Japan and Singapore have one patent each. France and Germany did not publish patents. Comparing the ratio of “emerging technology” of each country, China and India showed relatively high value: 24.2%, and 27.0%, respectively. Japan and Singapore did not publish patents in “emerging technology.” Both the USA and Australia showed intermediate values (16.7%). As above, country ranking in the number of patents is similar with that of academic papers. The percentage of “emerging technology” in patents is different from that in scientific papers, but this value of Japan, Australia, and Singapore is not useful due to a small number of patents.

**Table 6:** The number of patents in each class by countries.

Country	The number of patents (The percentage of publications in each technology by country)			
	Mature technology	Intermediate technology	Emerging technology	Total
China	691 (63.7%)	127 (11.7%)	266 (24.5%)	1,084 (100.0%)
USA	44 (73.3%)	6 (10.0%)	10 (16.7%)	60 (100.0%)
South Korea	28 (63.6%)	6 (13.6%)	10 (22.7%)	44 (100.0%)
India	18 (48.6%)	9 (24.3%)	10 (27.0%)	37 (100.0%)
Japan	1 (100.0%)	0 (0.0%)	0 (0.0%)	1 (100.0%)
France	0 (-%)	0 (-%)	0 (-%)	0 (-%)
Taiwan	- (-%)	- (-%)	- (-%)	- (-%)
Germany	0 (-%)	0 (-%)	0 (-%)	0 (-%)
Australia	3 (50.0%)	2 (33.3%)	1 (16.7%)	6 (100.0%)
Singapore	1 (100.0%)	0 (0.0%)	0 (0.0%)	1 (100.0%)

## 4 Discussion

Our study provides a new method to understand the interactions among three actors: science, technology, and research policy, for discussing an innovation strategy.

We observed science by clustering citation networks. Focusing on “electrode materials” knowledge domains, research related to various materials was observed such as composite materials containing carbon materials such as PANi/G, carbon materials like activated carbon, or metal oxides such as MnO_2_. The “Application” knowledge domains contained various applications including wearable SC and micro SC. The “others” knowledge domains mostly contained research on SC performance improvement except for electrode materials such as pore size in mesoporous carbon and ionic liquid. Our results imply that there are various knowledge domains in the SC research field, and most of them focus on electrode materials. Chen reported that there are five types of SC electrode materials: carbon materials, metal oxides, conducting polymers, nanocomposites, and carbon-polymer composites, and our method succeeded in finding these knowledge areas [63]. Thus, our method reflects the situation in SC research.

We assessed the maturity of knowledge domains using the developed matrix with two metrics. Regarding knowledge domains in “mature technology,” many have focused on carbon materials such as activated carbon and graphene. In contrast, “emerging technology” includes metal oxides/sulfides and MOF. “Intermediate technology” contains both carbon materials and metals. This result implies that the SC research trend has changed from carbon materials to metallic materials. Carbon materials and conducting polymers have been widely investigated and regarded as traditional electrode materials, and new electrode materials such as MOF have recently been introduced as frontrunners [64, 65]. Moreover, knowledge domains in “mature technology,” “intermediate technology,” and “emerging technology” tended to show a low, intermediate, and high growth rate of patents, respectively. It implies that using two metrics, in this case the average publication year and the number of nodes, is effective in evaluating the maturity of knowledge domains, and “emerging technology” is likely to have undergone rapid commercialization in the industry. As patents play an important role in commercializing scientific findings, our method provides a correlation between science and technology.

Next, we observed the research tendency in each country by calculating the percentage of publications in the “emerging technology” category. China and Singapore had high values, while, Japan, France, and Germany showed low ratios. Our results suggest that China and Singapore focus on “emerging technology,” whereas Japan, France, and Germany do not. We assumed that this difference was partly due to their electronic research policies and industrial strategies. To verify this, we investigated research policies and industrial events related to SC and compared them with the results.

China tends to focus on research into innovative SC. For example, the National Basic Research Program (Program 973) launched basic research on innovative batteries aiming to develop batteries with an energy density of more than 300 Wh/kg [66]. China conducts research mainly on large stationary energy battery storage, and there is relatively little research on lithium batteries. Focusing on the commercialization of batteries, more than half of China's urban population owns smart wristbands, and the market of wearable devices has been expanding rapidly [67, 68]. We assume that a result of this study that China published lots of patents in “emerging technology” reflects this social phenomenon. Meanwhile, Singapore, which has the highest ratio of “emerging technology,” emphasized on emerging technologies like nanoelectronics [69].

Europe including France and Germany emphasized graphene-based research and launched research policies related to graphene such as “Graphene Flagship (established in 2013)” and “Graphene 20XX (2011)” [70, 71]. This tendency is partially affected by Professor Andre Geim from Manchester University, who invented graphene in 2004, and won the Nobel Prize in 2010.

In the case of Japan, research concentrates not only on SC but also on other capacitors, such as lithium batteries and solid-state batteries, because Japanese research on batteries is mainly aimed at car batteries. Many projects related to lithium batteries and solid-state batteries such as ALCA-SPRING and Rising Ⅱ have been launched in recent years [72, 73]. Focusing on electrode materials, researchers in Japan made efforts in the field of organic electronics for many years [74, 75]. This is partly because Japanese chemists and winners of the Nobel Prize in Chemistry, Hideki Shirakawa and Akira Yoshino, invented conductive polymers and a lithium-ion battery, respectively [76, 77]. Japanese researchers began taking note of conductive polymer and lithium-ion batteries after the invention.

A similar tendency, seen in other research fields like stem cell research, is that Japan tends to be heavily influenced by the great discoveries made by Japanese researchers. Japanese researchers are prone to concentrate on iPS cells, whereas there are many more studies on ES cells than on iPS cells worldwide [78]. This is partly because the first discovery of iPS cells was by Shinya Yamanaka, a Japanese researcher who won the 2012 Nobel Prize in Physiology and Medicine. Since research on iPS cells accounts for a large portion of the Japanese regenerative medicine research budget, other regenerative medicine research fields should be enriched, and research policies that strengthen various regenerative treatments must be fulfilled. Even though the promotion of international collaborative research is important, the number of foreign researchers in Japan has declined since 2001 [47,79]. Thus, internationalization is an issue in Japanese research.

In contrast, Singapore has begun internationalizing research in various ways, for instance, by arranging a scholarship program and employing researchers from overseas [80]. Singapore has set up many scholarship systems for international students, such as the “National Science Scholarship.” Moreover, Nanyang Technological University has actively adopted foreign teachers for starting research activities and abolished a quarter of the teaching staffs’ lifetime employment rights. As a result, the percentage of research and development (R & D) foreigner workers in Singapore has increased to 32.0% [81]. This internationalization helps Singapore to obtain information from all over the world and catch up with the global research trends quicker than other countries. We assume that it leads to obtaining a higher percentage in emerging research technology areas.

As mentioned above, defining technology maturity area helped us observe academic research trend, detect rapidly commercializing technologies, and reflect countries’ research tendencies and policies. Knowledge domains in “emerging technology,” which has a small number of publications and low average publication year, showed a high increase rate of patents, whereas Singapore and China, which published many scientific papers in “emerging technology,” developed a research strategy superior in innovative technologies. These results imply the industry and government approach to new knowledge domains in academic research. Our method succeeded in obtaining the relationships among science, technology, and research policy, and detecting knowledge domains that are creating innovation.

## 5 Conclusion

The main purpose of this study was to examine the interactions among science, technology, and research policy at a knowledge domain level. Observing the relationships among three key actors is important for making an innovation strategy. However, in most cases, previous studies have only focused on university-industry collaboration. In addition, previous bibliometric research on triple helix did not analyze knowledge domains and only focused on the strength of industry-academia-government relationships. Thus, it was hard to find important knowledge domains for developing an innovation strategy. To solve this problem, we proposed a new method to evaluate the maturity of knowledge domains correctly and understand the interactions among science, technology, and research policy. In this study, we selected SC as a research target because SC has been attracting increasing attention in academic research, industrial businesses, and technology policies.

Our method consisted of the following three steps. First, we defined knowledge domains in SC by clustering a citation network of publications and recognized an overview of the scientific research field. These knowledge domains were classified into three groups by their research purposes: “electrode materials,” “applications,” and “others.” Second, we categorized knowledge domains using a developed matrix into three groups by their maturity; “mature technology,” “intermediate technology,” and “emerging technology.” Third, we examined the technology area each country focused on.

In the first step, we collected 28 knowledge domains. Because most of them were about electrode materials, various types of electrode materials for SC have been developed. In the second step, five of the knowledge domains were categorized as “emerging technology.” As these knowledge domains had a high growth rate of patents that were commercializing rapidly, we succeeded in predicting which research domains would be commercialized. In the third step, we analyzed each country's research tendencies by calculating the percentage of publications and patents in each technology maturity group. China and Singapore showed high rates, whereas Japan, France, and Germany had low values. This result reflects countries' research policies and implies that specialty research areas differed by country. For example, China has focused on innovative technologies, and Japan has been concentrating on mature technologies such as lithium-ion batteries. As above, our research result implies that academia, industry, and government have paid attention to knowledge domains in “emerging technology”, and these are important for creating innovation.

Our method is helpful to society in various ways. Academically, it can help researchers catch up with research trends identified in emerging research areas. From an industry perspective, this study can contribute to the discovery of developing technology areas expected to be commercialized early. It is important for companies that have the responsibility of providing new technology to society to find developing technology early, to commercialize it. From a policymaker's perspective, the study can aid government in evaluating the impact of research policy by calculating the share of developing research areas of each country. In addition, our method can be applied to evidence-based policymaking. Evidence-based policy is a term often used in various fields of public policy to refer to policy decisions informed by rigorously settled verifiable evidence. Good data and analysis methods are necessary to achieve devotion to scientifically appropriate practice [82, 83, 84].

Through this study, researchers, industrial companies, and policymakers could predict which knowledge domains in academic research would commercialize rapidly and be important to improve international competitiveness. As SC research experiences radical improvements, research strategies have become important for more papers in advanced research areas. The limitation of this study is obtaining the interaction between industry and government. Given that the increase rate of patents in “emerging technology” was high, our method revealed that the industry focuses on developing knowledge domains of academic research. In addition, this study evaluated the estimated effect of countries’ research policies by calculating the ratio of academic papers in each technology class. Our method succeeded in analyzing the relationships of academia-industry and academia-government as explained above but did not obtain the interaction between industry and government. Therefore, our future study will include patent analysis using the method proposed in this study and achieve a more accurate picture of the interactions, especially between industry and research policy [30].

## Declarations

### Author contribution statement

Chika Ishii: Conceived and designed the experiments; Performed the experiments; Analyzed and interpreted the data; Contributed reagents, materials, analysis tools or data; Wrote the paper.

Kimitaka Asatani: Performed the experiments; Analyzed and interpreted the data; Contributed reagents, materials, analysis tools or data.

Ichiro Sakata: Conceived and designed the experiments; Contributed reagents, materials, analysis tools or data.

### Funding statement

This work was supported by the SEUT RA program at the 10.13039/501100004721University of Tokyo.

### Data availability statement

Data included in article/supp. material/referenced in article.

### Declaration of interests statement

The authors declare no conflict of interest.

### Additional information

No additional information is available for this paper.

## Figures and Tables

**FigS1:**
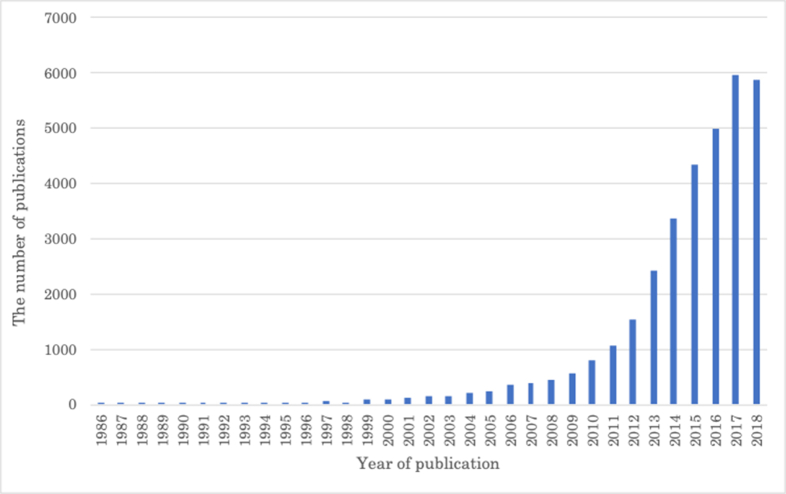
The number of the extracted publications per year

## Appendix A Supplementary data

SupplementalyInformation_20220816 SupplementalyInformation_20220816
