# Supplementary figures and images for: Obtaining interactions among science, technology, and research policy for developing an innovation strategy: A case study of supercapacitors
Source: Heliyon. 2022 Sep 21;8(9):e10721. doi: 10.1016/j.heliyon.2022.e10721 (PMC9526165; doi:10.1016/j.heliyon.2022.e10721)

SUPPLEMENTARY INFORMATION

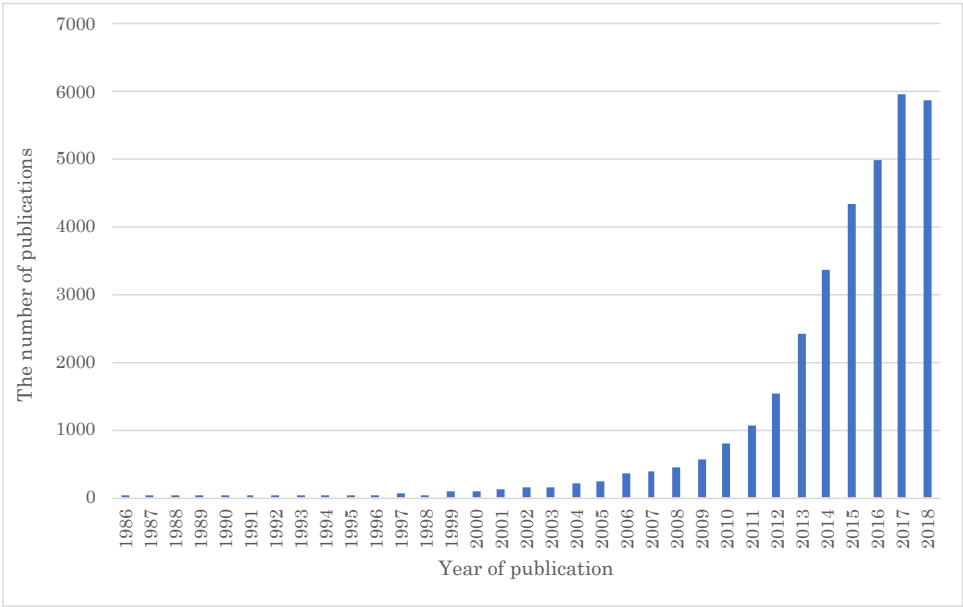

Figure S1. The number of the extracted publications per year

Supplement: SupplementalyInformation_20220816 [file mmc1.pdf]
